# Understanding PI-QUAL for prostate MRI quality: a practical primer for radiologists

**DOI:** 10.1186/s13244-021-00996-6

**Published:** 2021-05-01

**Authors:** Francesco Giganti, Alex Kirkham, Veeru Kasivisvanathan, Marianthi-Vasiliki Papoutsaki, Shonit Punwani, Mark Emberton, Caroline M. Moore, Clare Allen

**Affiliations:** 1 Department of Radiology, University College London Hospital NHS Foundation Trust, London, UK; 2 Division of Surgery and Interventional Science, University College London, London, W1W 7TS UK; 3 Department of Urology, University College London Hospital NHS Foundation Trust, London, UK; 4 Centre for Medical Imaging, University College London, London, UK

**Keywords:** Prostate cancer, Magnetic resonance imaging, Image quality, PI-QUAL score

## Abstract

Prostate magnetic resonance imaging (MRI) of high diagnostic quality is a key determinant for either detection or exclusion of prostate cancer. Adequate high spatial resolution on T2-weighted imaging, good diffusion-weighted imaging and dynamic contrast-enhanced sequences of high signal-to-noise ratio are the prerequisite for a high-quality MRI study of the prostate. The Prostate Imaging Quality (PI-QUAL) score was created to assess the diagnostic quality of a scan against a set of objective criteria as per Prostate Imaging-Reporting and Data System recommendations, together with criteria obtained from the image. The PI-QUAL score is a 1-to-5 scale where a score of 1 indicates that all MR sequences (T2-weighted imaging, diffusion-weighted imaging and dynamic contrast-enhanced sequences) are below the minimum standard of diagnostic quality, a score of 3 means that the scan is of sufficient diagnostic quality, and a score of 5 implies that all three sequences are of optimal diagnostic quality. The purpose of this educational review is to provide a practical guide to assess the quality of prostate MRI using PI-QUAL and to familiarise the radiologist and all those involved in prostate MRI with this scoring system. A variety of images are also presented to demonstrate the difference between suboptimal and good prostate MR scans.

## Key points

- PI-QUAL represents the first available scoring system to assess prostate MRI quality.
- PI-QUAL reinforces clinicians’ confidence in prostate MRI to determine patient care.
- PI-QUAL is the basis for future work and will undergo further refinements.

## Background

The evolution and rapid diffusion of prostate magnetic resonance imaging (MRI) has inevitably led to variability in vendor and scan quality among imaging centres across the world, with the high risk of generating images of suboptimal diagnostic quality [1–3].

We know that low diagnostic quality for some prostate MR images can reduce accuracy of prostate MRI, and limit confidence in the technique [4, 5]. In an attempt to address this, the Prostate Imaging Reporting and Data System (PI-RADS) standards for reporting set out the minimal technical requirements for the acquisition of multiparametric magnetic MRI (mpMRI) of the prostate throughout all updates since 2012 [6–8] (Table 1).

**Table 1:** Technical requirements for multiparametric prostate MRI according to PI-RADS v. 2.1 guidelines

	Imaging planes	Slice thickness	FOV	In-plane dimension	Specific recommendations		
T2w imaging	Same used for DWI and DCE	3 mm	12–20 cm^a^	≤ 0.7 mm (phase) × ≤ 0.4 mm (frequency)	Axial plane: either straight axial to the patient or in an oblique axial plane matching the long axis of the prostate	At least one additional orthogonal plane (sagittal and/or coronal)	3D axial as an adjunct to 2D acquisitions
		No gap					
DWI	Same used for T2w imaging and DCE	≤ 4 mm	16–22 cm	≤ 2.5 mm (phase and frequency)	Low *b* value: 50–100 s/mm^2^	Intermediate *b* value: 800–1000 s/mm^2^	High *b* value
		No gap					Dedicated (≥ 1400 s/mm^2^)
							Synthesised (from other *b* values)
DCE	Same used for T2w imaging and DWI	3 mm	No specific recommendations^a^	≤ 2 mm (phase and frequency)	Temporal resolution ≤ 15 s	GBCA: 0.1 mmol/kg	Fat suppression
		No gap				Injection rate: 2–3 cc/s	
						Observation rate ≥ 2 min	

*T2w imaging* T2-weighted imaging, *DWI* diffusion-weighted imaging, *DCE* dynamic contrast enhanced, *FOV* field of view, *GBCA* gadolinium-based contrast agent

^a^To encompass the entire prostate gland and seminal vesicles

Growing evidence that the quality of prostate MRI influences the rate of detection clinically significant prostate cancer has resulted in the publication of a number of studies addressing this topic [9–20]. In addition, two panels of experts [21, 22] have stressed the importance to establish quality criteria for the technical acquisition of mpMRI of the prostate.

A first attempt to address this topic has been the publication of the Prostate Imaging Quality (PI-QUAL) scoring system [23] from the multi-centre PRECISION trial [24].

The purpose of this educational review is to provide a practical guide to assess the quality of prostate MRI scans using the PI-QUAL score and to familiarise the radiologist and all those involved in prostate MRI with this dedicated scoring system.

In detail, we will cover each step that should be followed to assess imaging quality in a proper manner according to PI-QUAL. We will do so by providing examples of images of suboptimal versus adequate diagnostic quality.

At present, PI-QUAL represents the only available scoring system for evaluating the quality of prostate mpMRI scans so that the generalisability of results from multiple studies can be assessed.

Although there are some limitations (for example, we did not investigate how the quality of the dominant sequence should be weighted in the final assessment of the score), we believe that the results obtained from the widespread use of PI-QUAL will help the future iterations of this scoring system, which could include the extraction of objective quality metrics from the images (e.g. artificial intelligence models for scoring image quality).

While this guide is primarily intended for radiologists and trainees who are not very familiar with prostate MRI, this primer may be also useful for experienced radiologists working in academic/tertiary referral centres for prostate MRI in order to assess whether the quality of the scans performed outside of their institution is adequate or whether the scan should be repeated, before taking any clinical decisions (e.g. defer biopsy, MR-derived biopsy targets, treatment vs active surveillance).

## The PI-QUAL score

Since its publication in June 2020, the PI-QUAL score [23] has attracted some attention and comment [25, 26].

This scoring system has been created to assess the quality of mpMRI of the prostate against both a set of objective technical criteria (PI-RADS v. 2.0) [7] together with a set of subjective criteria from the MR images.

PI-QUAL is based on a 1-to-5 scale that indicates the adequacy of the diagnostic quality of a scan, where 1 indicates that all sequences [i.e. T2-weighted imaging (T2w imaging), diffusion-weighted imaging (DWI) and dynamic contrast enhanced (DCE) sequences] are below the minimum standard of diagnostic quality, 3 implies that the scan is of sufficient diagnostic quality, and 5 means that all three sequences are of optimal diagnostic quality. In particular, a PI-QUAL score ≥ 4 means that the quality of the MR is high, and all clinically significant lesions can be ruled in and out (Table 2).

**Table 2:** Assessment of the diagnostic quality of multiparametric MRI scans using the PI-QUAL score

PI-QUAL score	Criteria	Clinical implications
1	All mpMRI sequences are below the minimum standard for diagnostic quality	It is NOT possible to rule in all significant lesions^a^
2	Only one mpMRI sequence is of acceptable diagnostic quality	It is NOT possible to rule out all significant lesions^a^
3	At least two mpMRI sequences taken together are of diagnostic quality	It is possible to rule in all significant lesions
		It is NOT possible to rule out all significant lesions
4	Two or more mpMRI sequences are independently of diagnostic quality	It is possible to rule in all significant lesions
5	All mpMRI sequences are of optimal diagnostic quality	It is possible to rule out all significant lesions

*PI-QUAL* Prostate Imaging Quality, *mpMRI* multiparametric magnetic resonance imaging, *PI-RADS* Prostate Imaging Reporting and Data System

Reprinted with permission from Giganti et al. [23]

^a^Therefore, reports should not include PI-RADS or Likert scores

**Fig. 1:**
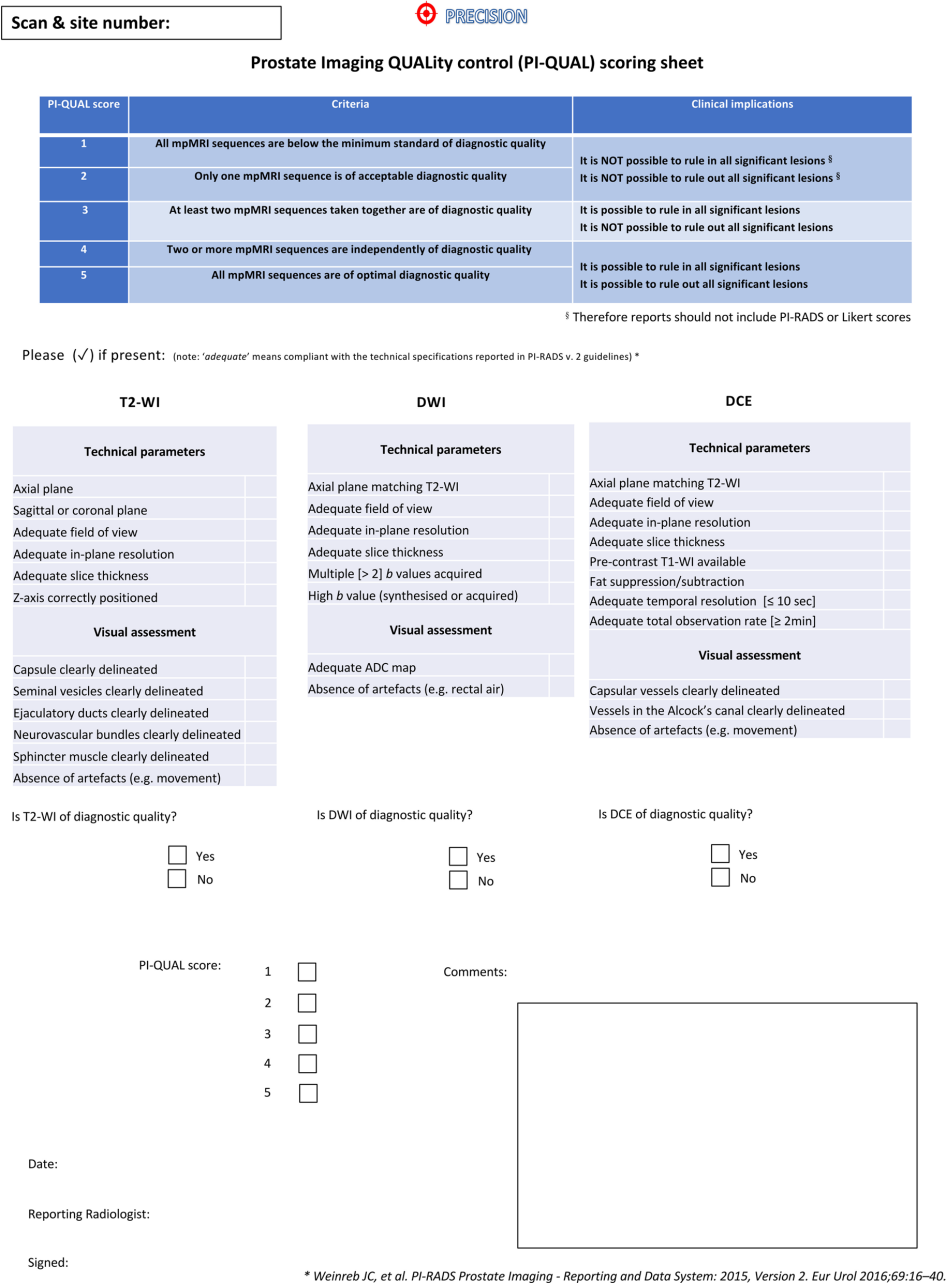
Scoring sheet for assessing the quality of multiparametric magnetic resonance imaging using the PI-QUAL score. *T2-WI* T2-weighted imaging, *DWI* diffusion-weighted imaging, *DCE* dynamic contrast enhanced, *ADC* apparent diffusion coefficient. Reprinted with permission from Giganti et al. [23]

The original document outlining the PI-QUAL score includes a dedicated scoring sheet that incorporates the technical parameters and the visual evaluation to be checked for each single MR sequence before assessing the PI-QUAL score (Fig. 1).

**Fig. 2:**
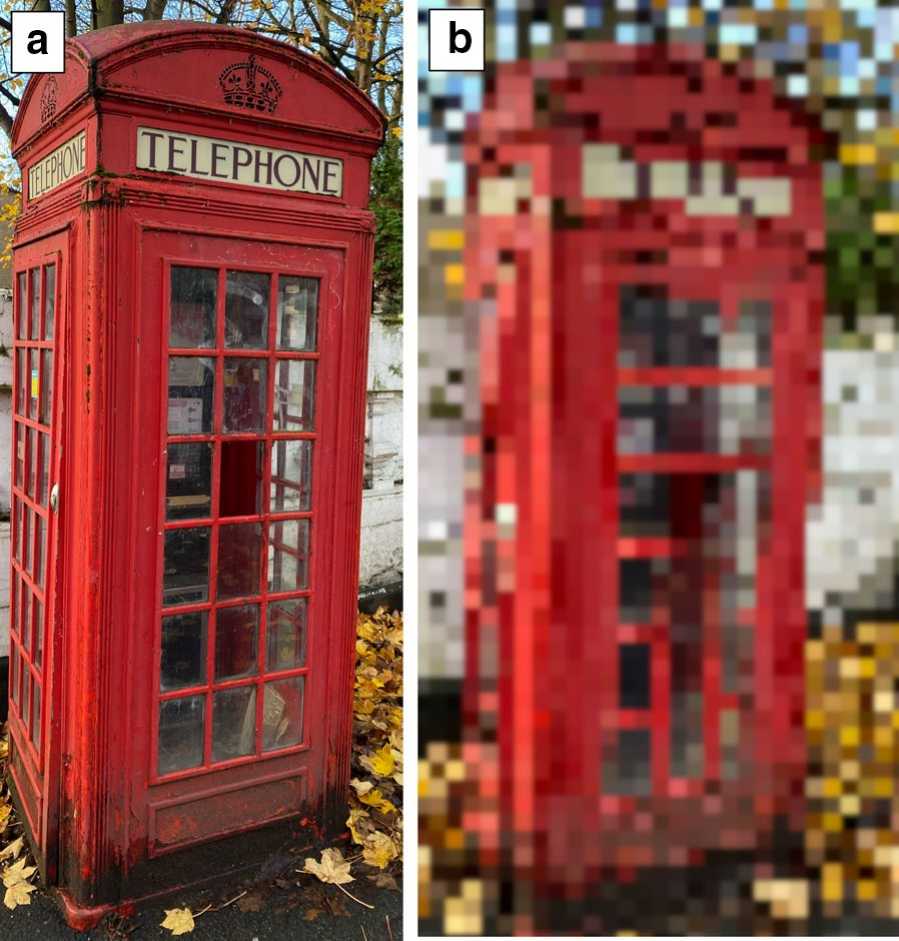
An example of the same image with different pixel sizes. Increasing pixel size from (**a**) to (**b**) reduces the image resolution

**Fig. 3:**
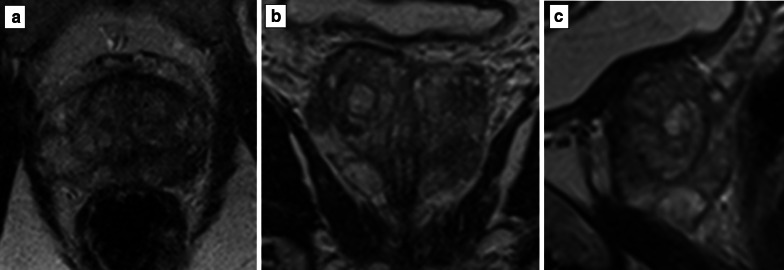
Axial (**a**), coronal (**b**) and sagittal (**c**) T2-weighted magnetic resonance imaging acquisitions of the same patient. Each acquisition was 4 min long, but the pixel size varied for each plane. The pixel size was 0.60 x 0.60 mm in (**a**), 0.67 x 0.67 mm in (**b**) and 0.80 x 0.80 mm in (**c**). This image shows that as the pixel size goes up, there is less noise but also that sharpness is reduced

Before discussing each single item included in the PI-QUAL scoring sheet, some basic concepts need to be mentioned in order to understand the problems that could affect image quality in prostate MRI:

- *Field of view* (FOV): determines the amount of coverage of the object of interest that we have in each plane.
- *Pixel*: the smallest 2D element in an image with dimensions along two directions, phase encoding and frequency encoding. The pixel size determines the trade-off between resolution and signal-to-noise ratio (SNR): increasing pixel size reduces resolution and increases SNR for a given scan time (Fig. 2).
- *Voxel*: the 3D volume element whose dimensions are given by the pixel together with the slice thickness (i.e. the measurement along the third axis).
- *Image Matrix* defines the number of rows and columns in the image, corresponding to the frequency and phase encoding directions.
- *In-plane (spatial) resolution:* determined by the pixel size (FOV/matrix). For the same matrix the image resolution will be inversely proportional to the FOV. Reducing FOV will therefore increase resolution but will also reduce the image SNR unless other parameters are altered to compensate (Fig. 3). Wrap artefact can occur due to aliasing (i.e. the structures that lie beyond the edges of the FOV are projected onto the other side of the image).
- *Slice thickness:* is an important factor for the resolution of the images and is strictly linked to the *slice increment.* The slices should be contiguous but are often obtained with a slice gap in order to increase the SNR. The gap is often 10% of the slice thickness and there will be no information from the missed section, so anatomical information or objects might not be included in the scan.
- *Artefacts:* these are image features caused by a variety of factors that can be related to patients (e.g. motion, rectal air or metallic implants) or MR scanners (e.g. hardware or software). Each artefact has a characteristic appearance that can be easily identified with experience.

We will now discuss each item of the original PI-QUAL scoring sheet for each sequence (T2w imaging, DWI and DCE) making reference to the latest version of the PI-RADS guidelines (v. 2.1) [8] for this primer and include relevant images of suboptimal versus optimal quality when necessary to show the reader how to assess imaging quality in a proper manner.

As outlined in the PI-RADS v. 2.1 recommendations [8], the fundamental advantage of 3 T compared with 1.5 T lies in an increased SNR, which increases linearly with the static magnetic field. This may be exploited to increase spatial resolution, temporal resolution or both.

Although artefacts related to susceptibility, and signal heterogeneity can also increase at 3 T, current 3 T scanners can address these issues in a variety of ways and the difference is often not marked.

Other factors can affect image quality besides magnetic field strength, and both 1.5 T and 3 T can provide adequate and reliable diagnostic examinations when acquisition parameters are well optimised. However, some of the PI-RADS recommendations are difficult to be met at 1.5 T and most members of the PI-RADS Steering Committee recommend 3 T for prostate MRI, although in the presence of implanted devices (e.g. metallic hip prosthesis), 1.5 T scanners may sometimes produce a more diagnostic image because of reduced artefact.

In addition to this, the use of coils can impact image quality. We know that endorectal coils increase SNR in the prostate at any magnetic field strength and this may be particularly valuable for inherently lower SNR sequences, such as DWI. However, a misplaced endorectal coil can cause severe artefacts that can impair the ability to correctly identify prostate cancer in the posterior gland. Moreover, the PI-RADS v. 2.1 recommendations clearly state that there are many other technical factors that influence SNR (e.g. receiver bandwidth, coil design, efficiency of the radiofrequency chain), and some 1.5 T scanners that employ a high number of external phased array coil elements and radiofrequency channels (i.e. ≥ 16) may achieve adequate SNR without an endorectal coil.

### T2-weighted imaging

T2w imaging is useful to study the anatomy of the prostate and surrounding structures and is the dominant sequence for the transition zone.

Prostate cancer is hypointense on T2w imaging.

There has been a lot of interest in the use of 3D axial acquisitions as an adjunct to 2D acquisitions. If acquired using isotropic voxels, 3D acquisitions may be particularly useful for a detailed visualisation of the anatomy and for the segmentation before MRI-fusion biopsies. However, it should be acknowledged that, in some cases, the contrast resolution and the in-plane resolution can be inferior to 2D T2w imaging.

- *Axial, sagittal and coronal planes*

The axial T2w imaging depicts the prostate zonal anatomy and its relationship to the urethra and is useful to evaluate extra-prostatic extension. The evaluation on T2w imaging is based both on signal intensity and morphology (e.g. encapsulation).

The urethra, verumontanum and the levator ani can be seen in their long axes on the coronal acquisition. The sagittal plane can be used to establish the relationship between the bladder, prostate and rectum.

**Fig. 4:**
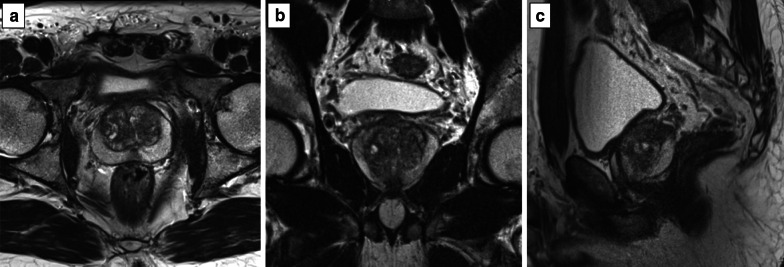
T2-weighted images obtained in the axial (**a**), coronal (**b**) and sagittal (**c**) planes

According to the PI-RADS v. 2.1 recommendations [8], T2-weighted images should always be obtained in the axial plane (either straight axial to the patient or in an oblique axial plane matching the long axis of the prostate) and a minimum of one additional orthogonal plane (i.e. sagittal and/or coronal), as shown in Fig. 4.

The acquisition of at least two planes facilitates the assessment of the morphology of anatomical structures and lesions (e.g. encapsulation) that could otherwise be limited by volume averaging if using a single plane, and it is also helpful to assess the degree of extraprostatic extension (e.g. seminal vesicle involvement) when present.

**Fig. 5:**
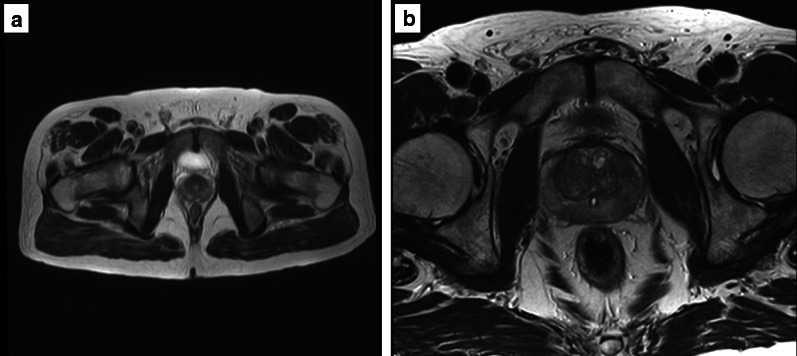
T2-weighted axial images of a suboptimal (41 × 42 cm) (**a**) and optimal (17 × 18 cm) (**b**) field of view according to the PI-RADS v.2.1 guidelines

**Fig. 6:**
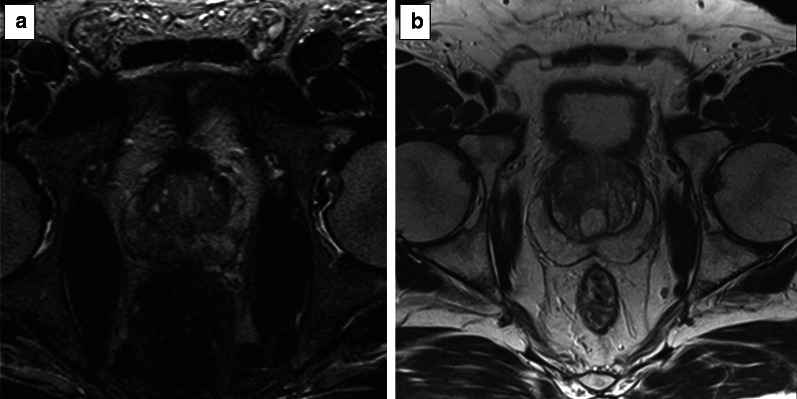
T2-weighted axial images of suboptimal (**a**) and optimal (**b**) in-plane resolution

**Fig. 7:**
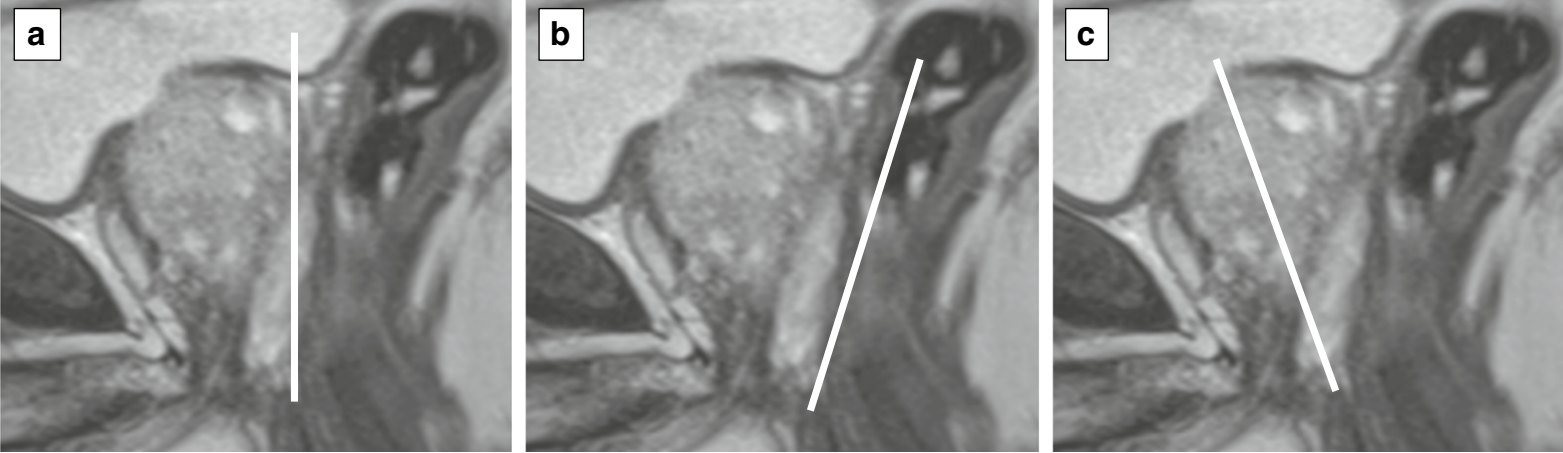
Sagittal T2-weighted acquisitions showing different positions of the axial plane: perpendicular to the MR table/patient (**a**), orthogonal to the rectum and posterior aspect of the prostate (**b**) or to the long axis of the prostate (**c**)

- *Field of view:* the FOV of T2w imaging should range from 12 to 20 cm to encompass the entire prostate gland and seminal vesicles (Fig. 5).
- *In-plane resolution:* the in-plane dimensions on T2w imaging should be ≤ 0.7 mm (for phase) × ≤ 0.4 mm (for frequency) (Fig. 6)
- *Slice thickness:* the slice thickness for T2w imaging should be 3 mm with no gap.
- *Z-axis:* with the patient supine in the MR scanner, the *z*-axis begins from the patient’s feet to head, the *y*-axis from dorsal to ventral and the *x*-axis from left to right. By convention, the direction of the main magnetic field is designated to be the *z*-axis. The position of the axial plane can vary between institutions (e.g. perpendicular to the MR table/patient, orthogonal to the rectum or in an oblique axial plane matching the long axis of the prostate) (Fig. 7).
- *Anatomical structures*

The delineation of anatomical structures on T2w imaging scans can be used as an objective marker of scan quality.

**Fig. 8:**
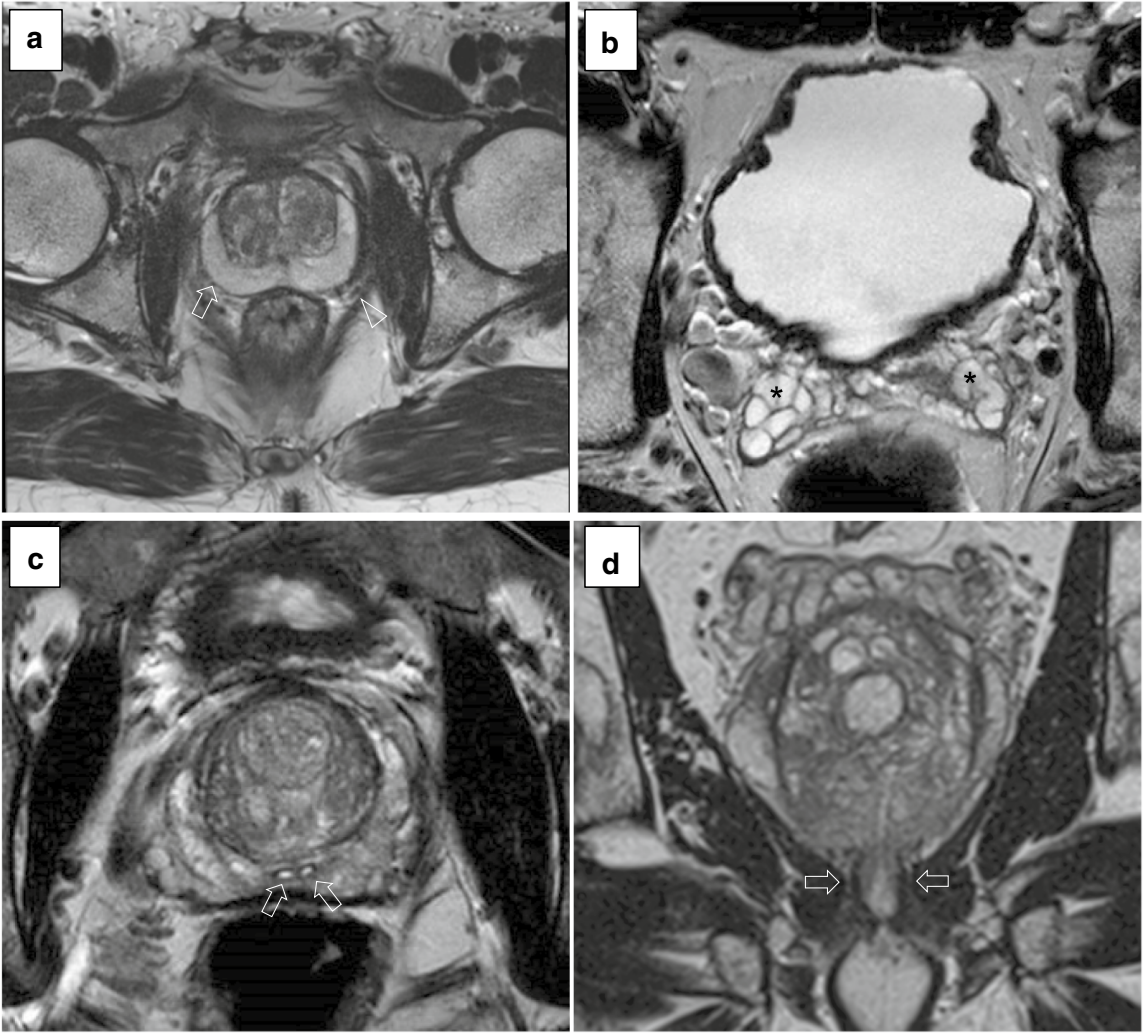
Axial (**a**–**c**) and coronal (**d**) T2-weighted images of good quality showing the prostatic capsule (**a**, arrow), the neurovascular bundle (**a**, arrowhead), the seminal vesicles (**b**, asterisks), the ejaculatory ducts (**c**, arrows) and the external sphincter (**d**, arrows), as mentioned in the visual assessment of the PI-QUAL scoring sheet

Figure 8 shows the five anatomical structures (i.e. capsule, neurovascular bundles, seminal vesicles, ejaculatory ducts and the external sphincter) mentioned in the visual assessment of the PI-QUAL scoring sheet. Clear visualisation of these structures is needed for the T2-weighted scans to be used to identify tumours within the prostate and for staging.

**Fig. 9:**
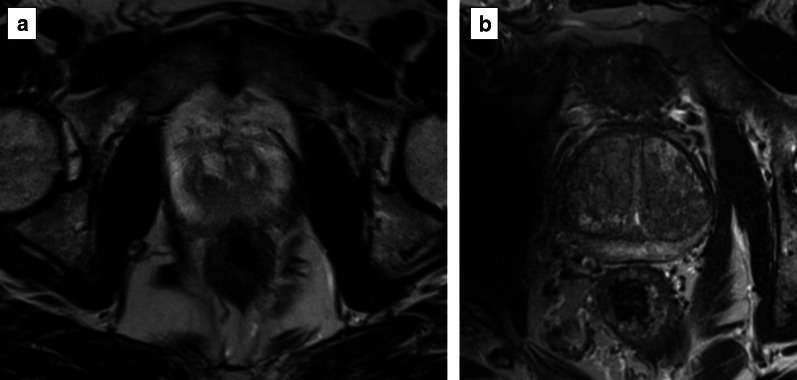
Axial T2-weighted images showing artefacts from movement (**a**) and metallic implants in the right hip (**b**)

- *Artefacts:* the most common artefacts on T2w imaging are caused by patient’s movement and by metallic implants, as shown in Fig. 9.

### Diffusion-weighted imaging

DWI reflects the random motion of water molecules (Brownian motion) and is a key component of prostate mpMRI. It should include an apparent diffusion coefficient (ADC) map extrapolated by multiple *b* values and a separate high *b* value diffusion-weighted acquisition.

Areas of restricted diffusion due to the high cellularity (as in prostate cancer) are hyperintense on the high *b* value and hypointense on the ADC map acquisition.

**Fig. 10:**
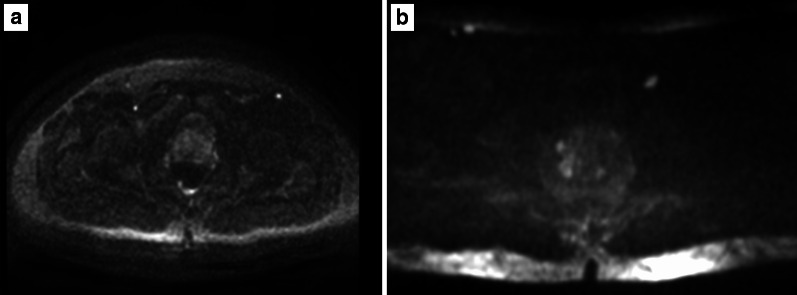
DWI of a suboptimal (38 × 40 cm) (**a**) and optimal (17 × 20 cm) (**b**) field of view according to the PI-RADS v.2.1 guidelines

**Fig. 11:**
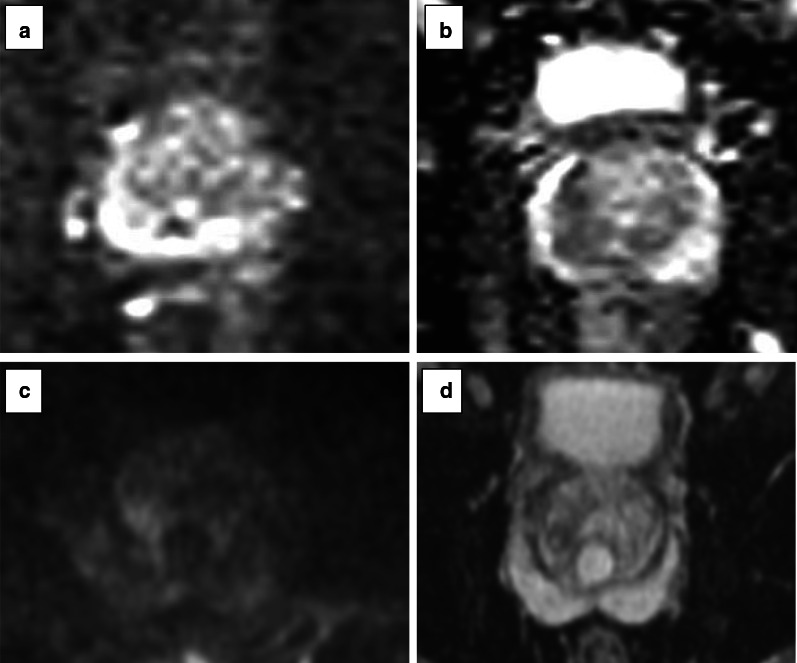
DWI of suboptimal (**a**, **b**) and optimal (**c**, **d**) in-plane resolution for the high b sequence (**a**, **c**) and ADC map (**b**, **d**), respectively

- *Field of view:* the FOV of DWI should range from 16 to 22 cm (Fig. 10).
- *In-plane resolution:* the in-plane dimensions on DWI should be ≤ 2.5 mm both for phase and frequency) (Fig. 11).
- *Slice thickness:* the slice thickness for DWI should be ≤ 4 mm with no gap.
- *Multiple b values*

Multiple *b* values should be acquired, but if only two can be obtained due to time or scanner constraints, it is recommended to use one low (preferably 50–100 s/mm^2^) and one intermediate *b* value (800–1000 s/mm^2^).

**Fig. 12:**
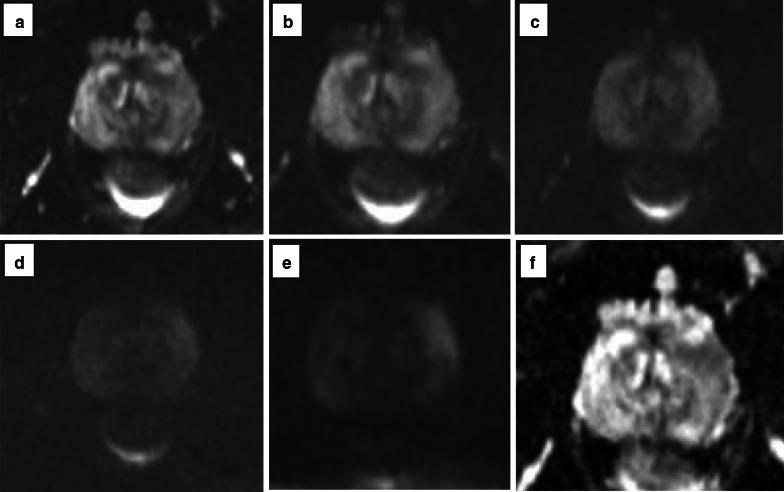
DWI at different *b* values (0–150–500–1000 s/mm^2^) in **a**–**d**, respectively. Dedicated high *b* sequence (*b* = 1400 s/mm^2^) and corresponding ADC map on a 1.5 T MR system in **e** and **f**, respectively

The maximum *b* value to calculate the ADC is recommended to be ≤ 1000 s/mm^2^ to avoid diffusion kurtosis effect (Fig. 12).

- *High b value sequence*

High *b* value images can be obtained directly by acquiring a high *b* value sequence (≥ 1400 s/mm^2^, requiring additional scan time) or calculated (i.e. synthesised) from the low and intermediate *b* value images to create the ADC map (this approach is less prone to artefacts because there is no need of longer echo times that are required for the high *b value* acquisition) [8]. The choice between a dedicated or synthesised high *b* value is still a matter of debate. It should be also mentioned that as the *b* value increases, the SNR decreases, so that the magnetic field strength and software used play an important role on DWI (Fig. 12).

- *Adequate ADC map*

The ADC map computes ADC values on a pixel-by-pixel basis using data from raw data sets obtained with different *b* values. Higher ADC values (i.e. low restriction of the diffusion) are hyperintense, while lower ADC values (i.e. high restriction of the diffusion) are hypointense on the ADC map.

**Fig. 13:**
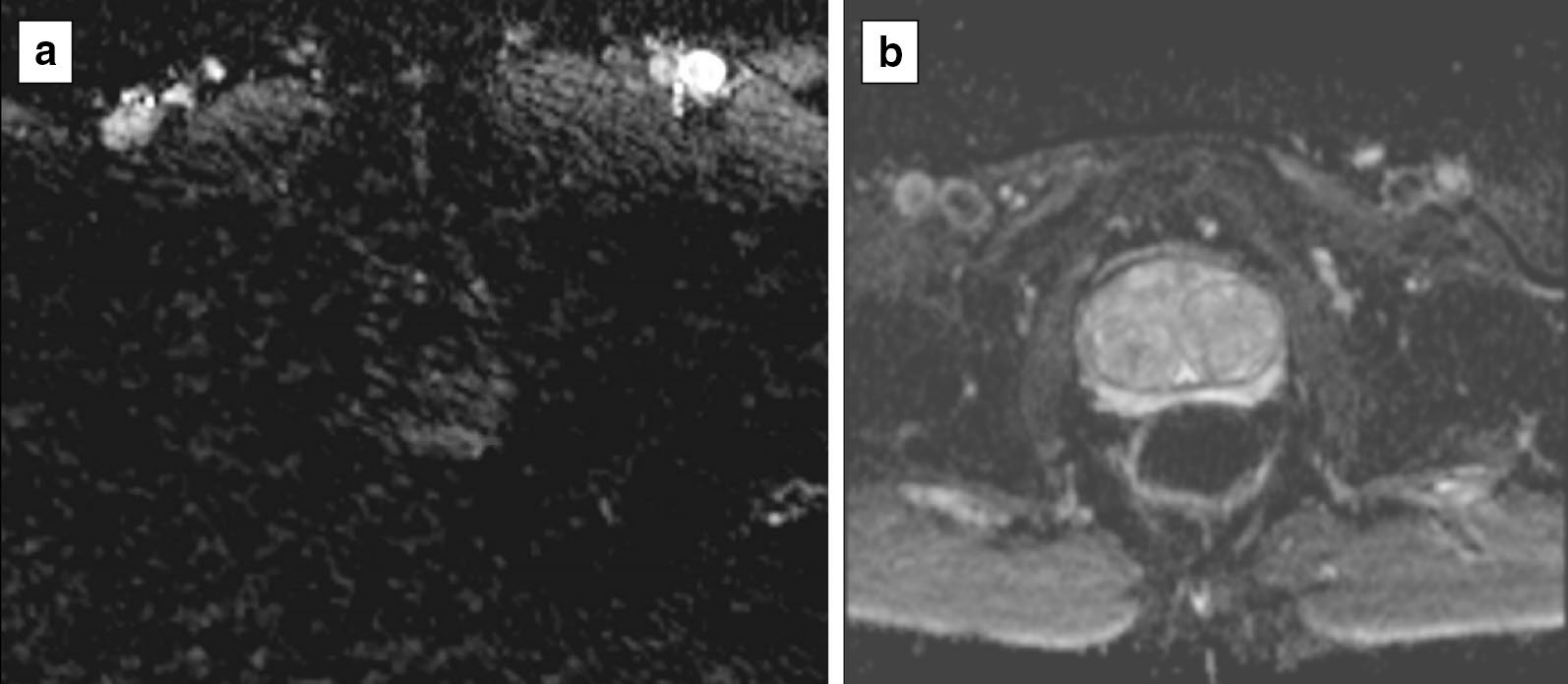
ADC maps of suboptimal (**a**) and optimal (**b**) quality

Examples of poor- and good-quality ADC maps are presented in Fig. 13.

**Fig. 14:**
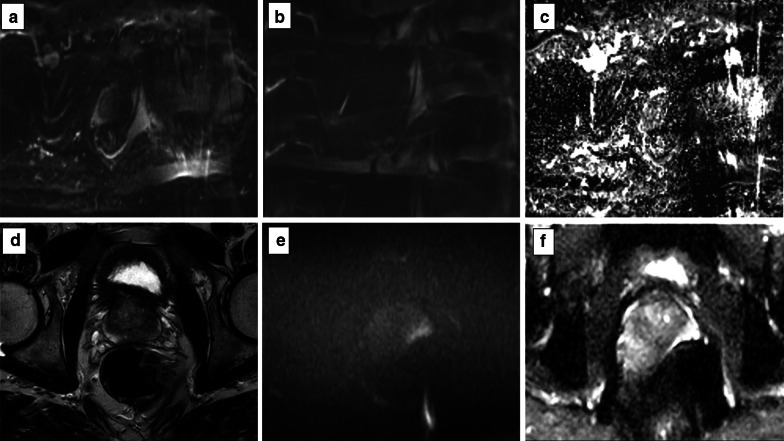
DWI hampered by artefacts from metallic implants (left hip) (*b* = 150 and 1400 s/mm^2^ in **a**, **b**, respectively, and ADC map in **c**) and rectal air (T2w imaging) in **d** showing the distended rectum, *b* = 1400 s/mm^2^ and ADC map in **e**, **f**, respectively)

- *Artefacts:* the most common artefacts on DWI are caused by metallic implants and rectal air, as shown in Fig. 14.

### Dynamic contrast-enhanced sequences

DCE-MRI refers to the rapid serial acquisition of T1-weighted gradient echo scans before, during and after the intravenous administration of a low molecular weight gadolinium-based contrast agent. Prostate cancer shows early enhancement and early washout due to increased vascularity and angiogenesis, and the use of contrast is particularly useful when T2w imaging and DWI are equivocal or degraded by artefacts.

**Fig. 15:**
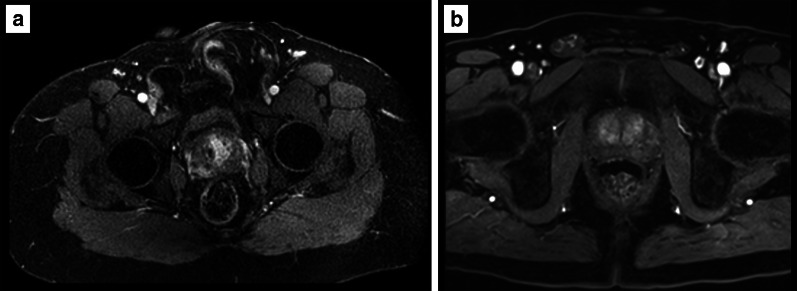
DCE of a suboptimal (36 × 40 cm) (**a**) and optimal (22 × 20 cm, encompassing the entire prostate gland and seminal vesicles—not shown) (**b**) field of view according to the PI-RADS v. 2.1 guidelines. Note the left inguinal hernia containing bowel loops in **a**

**Fig. 16:**
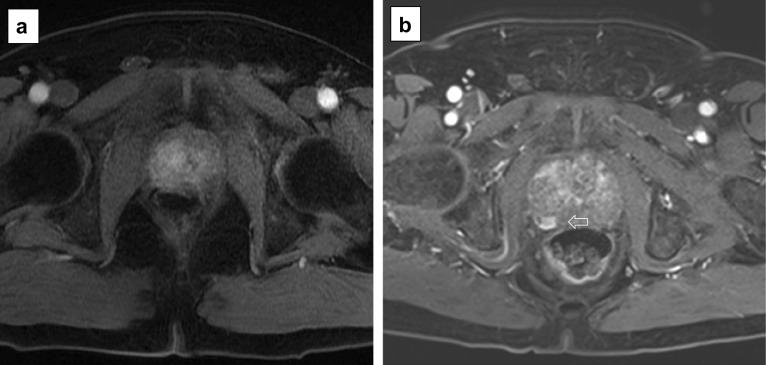
DCE of suboptimal (**a**) and optimal (**b**) in-plane resolution. The arrow in (**b**) indicates an enhancing lesion in the right peripheral zone (Gleason 4 + 3 at targeted biopsy)

- *Field of view:* according to PI-RADS v. 2.1 guidelines [8], the FOV for DCE sequences should encompass the entire prostate gland and seminal vesicles but should not be too large; otherwise, the spatial resolution could be impaired (Fig. 15).
- *In-plane resolution:* the in-plane dimensions on DCE should be ≤ 2 mm both for phase and frequency (Fig. 16).
- *Slice thickness:* the slice thickness for DCE should be 3 mm with no gap and should match the position of the T2-weighted axial scans.
- *Pre-contrast T1-WI available*

**Fig. 17:**
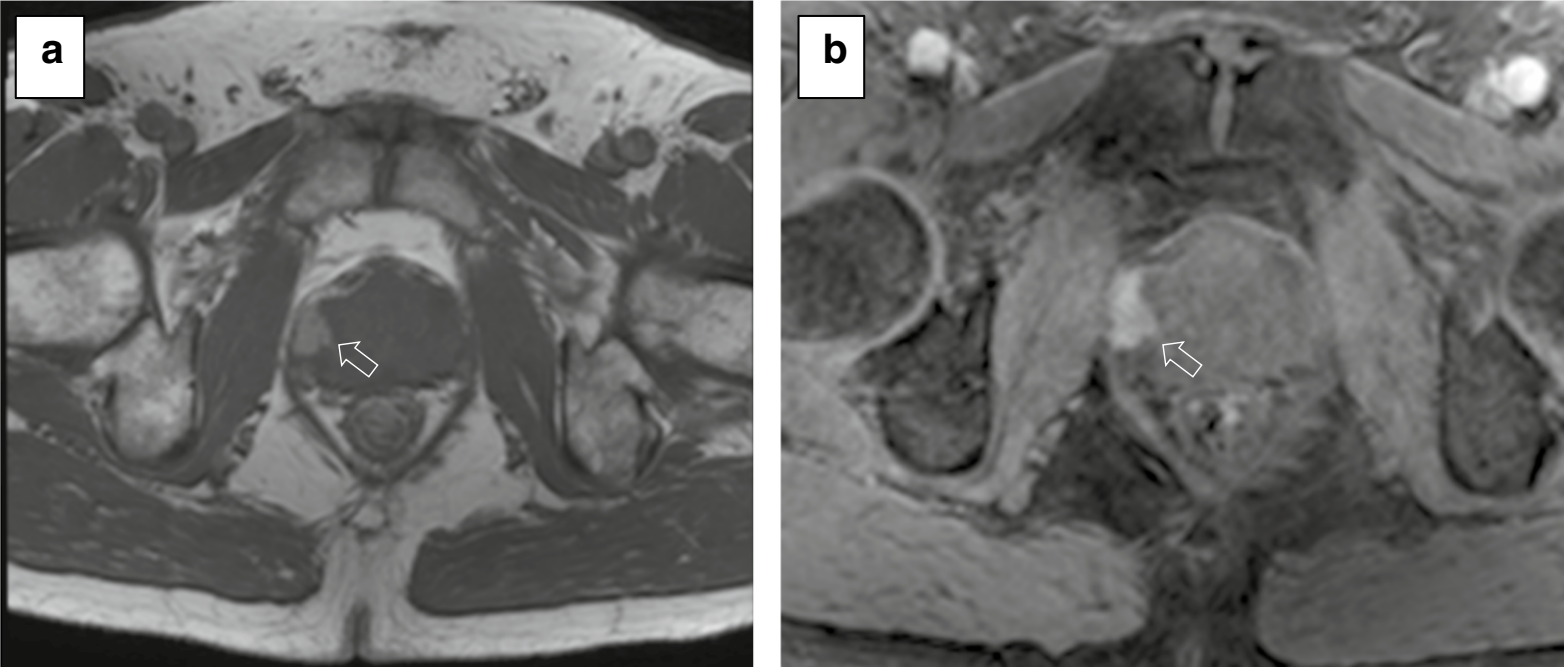
Pre-contrast non-fat suppressed (**a**) and fat-suppressed (**b**) T1-weighted images showing a hyperintense focus in the right anterior horn (arrows) in keeping with post-biopsy haemorrhage

Pre-contrast T1-WI is of utmost importance to rule out post-biopsy changes (e.g. haemorrhage), which impact adversely the quality of prostate MRI, especially for staging. These are seen as hyperintense areas in the pre-contrast T1 acquisitions. The PI-RADS guidelines recommend an interval of at least 6 weeks (or longer) after biopsy before performing the scan (Fig. 17). It is essential that this sequence is assessed for diagnostic quality prior to the injection of gadolinium and the initial images of a fat-suppressed dynamic study can represent a valid alternative to a dedicated pre-contrast T1 acquisition, reducing the total duration of the MR study.

It should be also noted that mpMRI is increasingly being performed in biopsy-naïve patients and therefore post-biopsy artefacts are now becoming less common.

- *Temporal resolution*

The temporal resolution provides information on the distance of time between the acquisitions of two images of the same area. The higher the temporal resolution, the shorter the acquisition of DCE images. DCE sequences of the prostate MR are generally acquired continuously for several minutes in order to detect early enhancing lesions in comparison with background prostatic tissue.

**Fig. 18:**
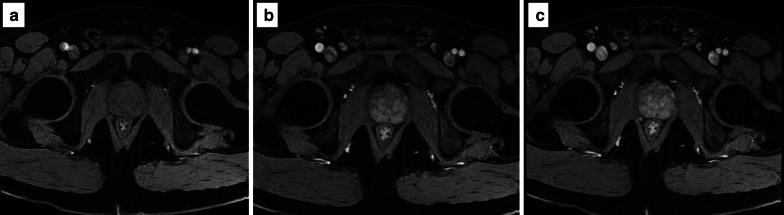
Three sequential DCE acquisitions with a temporal resolution of 30 s (i.e. 30 s between each acquisition). In this case it would be impossible to depict any focal early enhancement, which is characteristic of prostate cancer, in particular between **a** and **b**

As per PI-RADS v. 2.1 guidelines [8], temporal resolution should be  ≤ 15 s in order to depict focal early enhancement and early washout, which is characteristic of prostate cancer as previously mentioned (Fig. 18).

There is a balance between spatial and temporal resolution, and it is essential that this is balanced to produce scans that allow visualisation of early focal enhancement with good plane resolution.

- *Fat suppression*

**Fig. 19:**
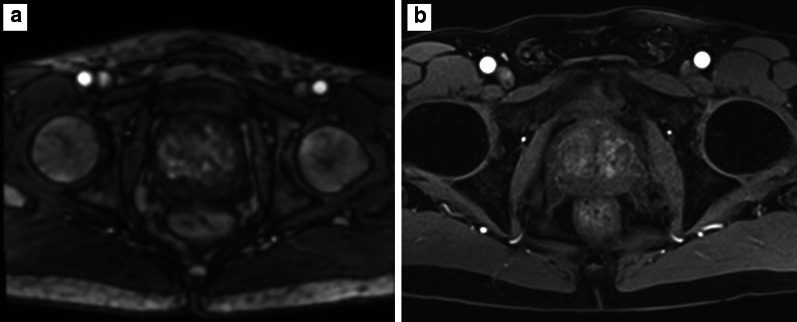
Non-fat suppressed (**a**) and fat-suppressed (**b**) DCE acquisitions

The PI-RADS v. 2.1 guidelines [8] recommend fat-suppression techniques, as the visual assessment of enhancement is improved (especially in the presence of post-biopsy artefacts that are hyperintense on T1-weighted imaging) and the capsule is better defined. Signal from adipose tissue can be suppressed using different techniques, which include saturation, short tau inversion recovery (STIR) sequences or the Dixon technique. Examples of non-suppressed and suppressed T1-weighted images are presented in Fig. 19.

- *Capsular vessels and pudendal artery*

**Fig. 20:**
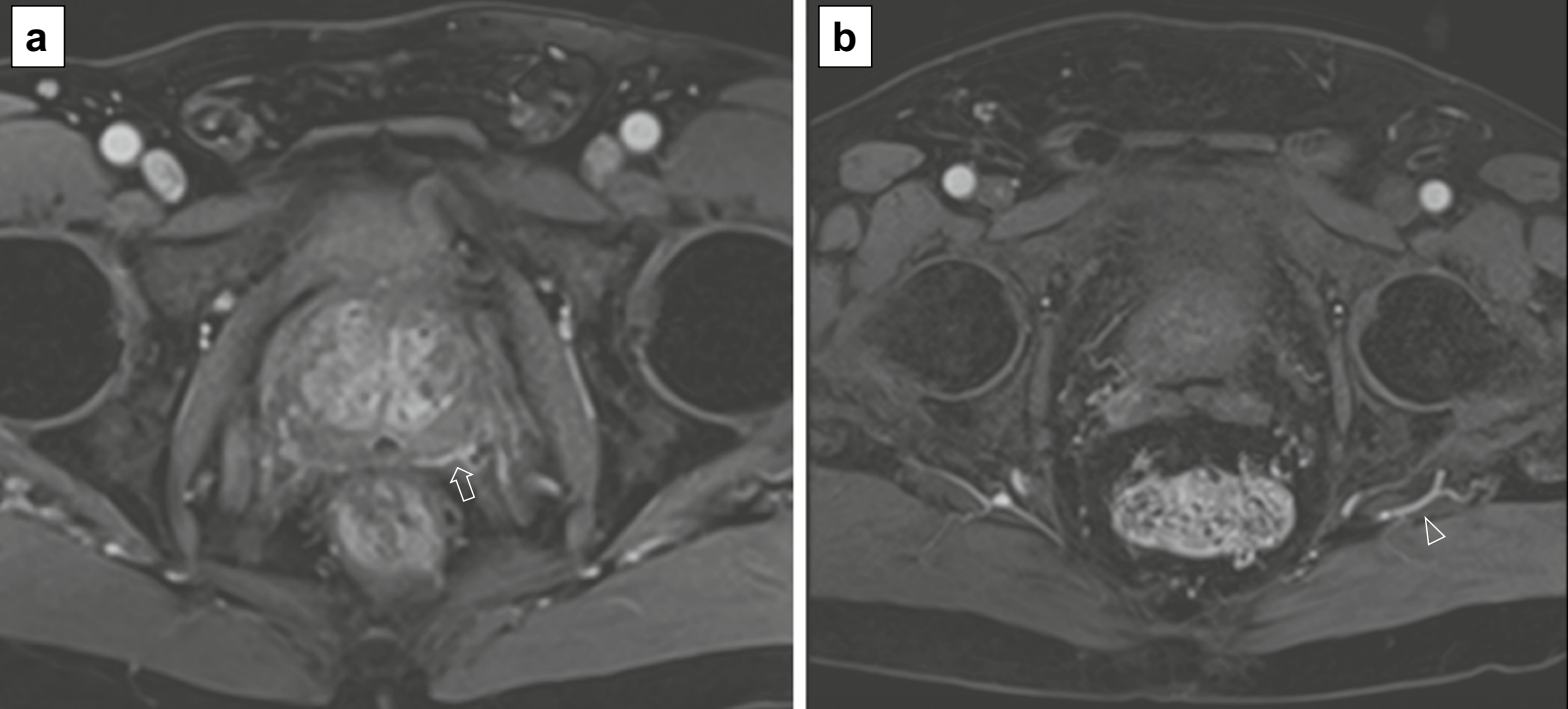
DCE images of adequate diagnostic quality showing the capsular vessels (**a**, arrow) and the vessels in the Alcock’s canal (**b**, arrowhead)

The visualisation of small blood vessels near the prostate can be used as an objective marker of scan quality. Various blood vessels can be used for this: prostate capsular vessels and the Alcock’s (or pudendal) canal (in which the internal pudendal artery, internal pudendal veins and the pudendal nerve pass). These are assessed on the PI-QUAL scoring sheet, as shown in Fig. 20.

**Fig. 21:**
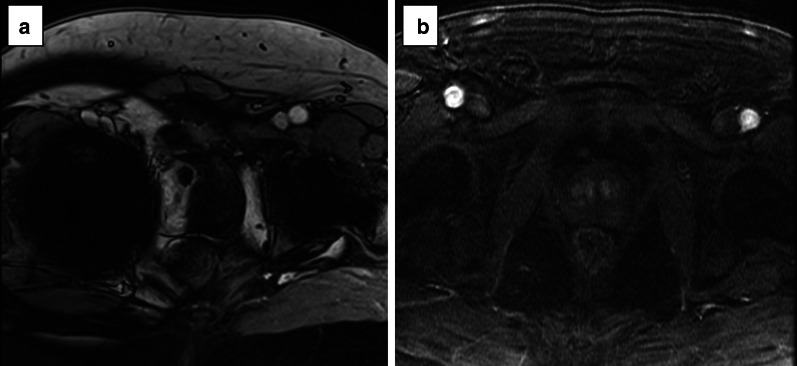
DCE images hampered by artefacts from metallic implants in the right hip (**a**) and from patient’s movement (**b**)

- *Artefact:* the most common artefacts on DCE are caused by metallic implants, poor fat suppression and patient’s movement, as shown in Fig. 21.

We will now present five different sets of images, one for each PI-QUAL score.

**Fig. 22:**
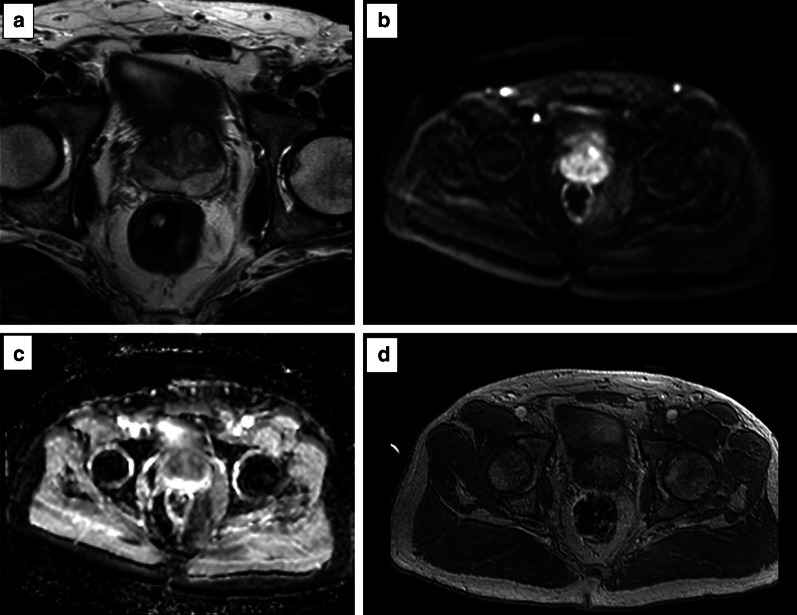
Axial T2w imaging (**a**), DWI with a *b* value of 150 s/mm^2^ (**b**), ADC map (**c**) and DCE acquisition (**d**) of a study that was given a PI-QUAL score of 1. All MR sequences are below the minimum standard of diagnostic quality as per PI-RADS v.2.1 technical recommendations. In particular, T2w imaging and DWI (**a**–**c**) show motion artefacts, no high *b* value has been acquired (**b**), the field of view is too large on DWI (21 × 35 cm, in **b**, **c**) and on DCE sequences (21 × 33 cm in **d**), and there is no fat suppression of DCE sequences (**d**)

**Fig. 23:**
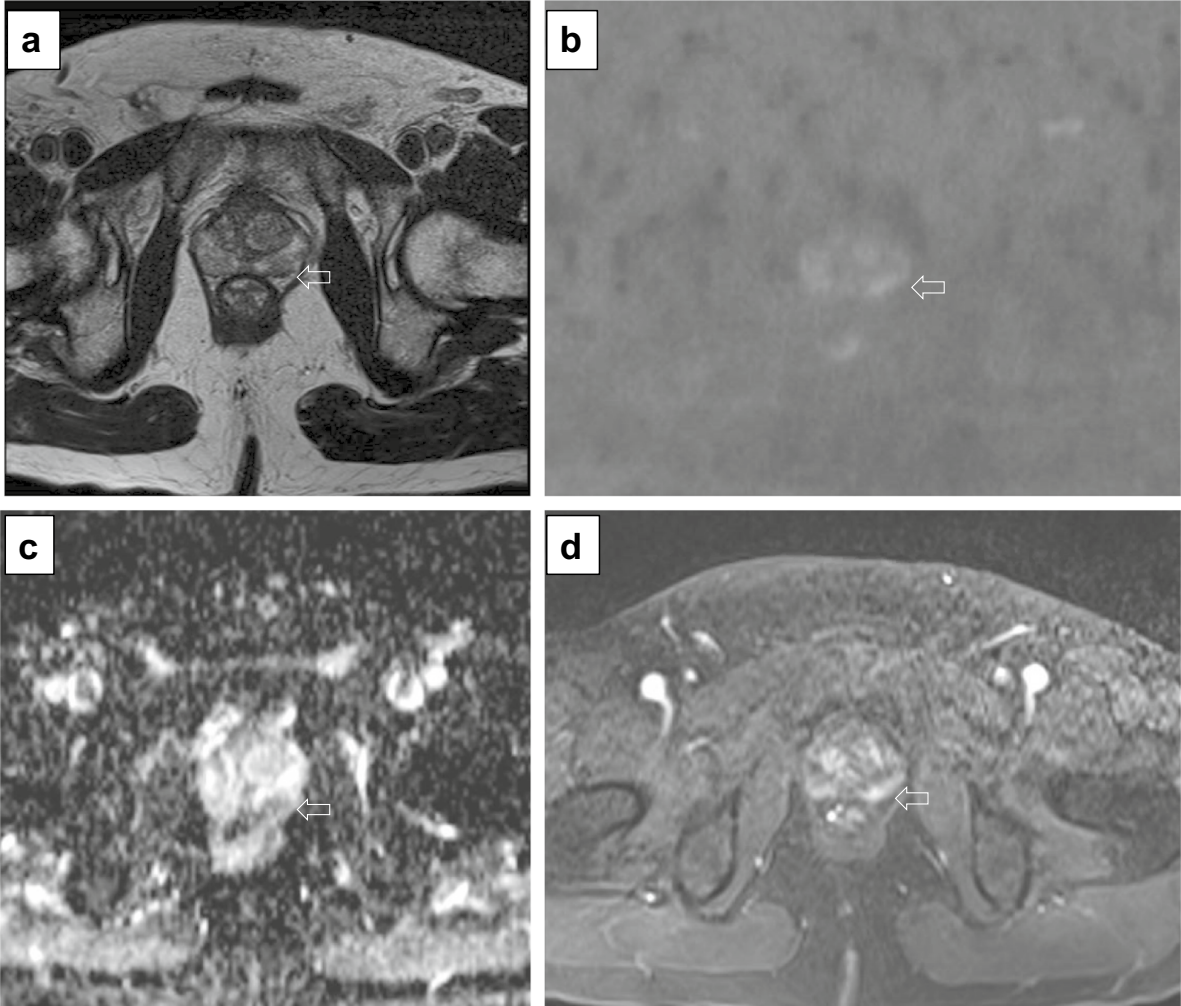
Axial T2w imaging (**a**), high *b* value (**b**), ADC map (**c**) and DCE acquisition (**d**) of a study that was given a PI-QUAL score of 2. Only T2w imaging (**a**) is of acceptable diagnostic quality (although the slice thickness is 3.5 mm). The in-plane resolution (including the ADC map) and the slice thickness (5 mm) of DWI and the in-plane resolution and slice thickness (3.5 mm) of DCE sequences are below the minimum standard of diagnostic quality as per PI-RADS v.2.1 technical recommendations. The arrows indicate a lesion in the left peripheral zone between 5 and 6 o’clock. Targeted biopsy revealed Gleason 3 + 4 disease

**Fig. 24:**
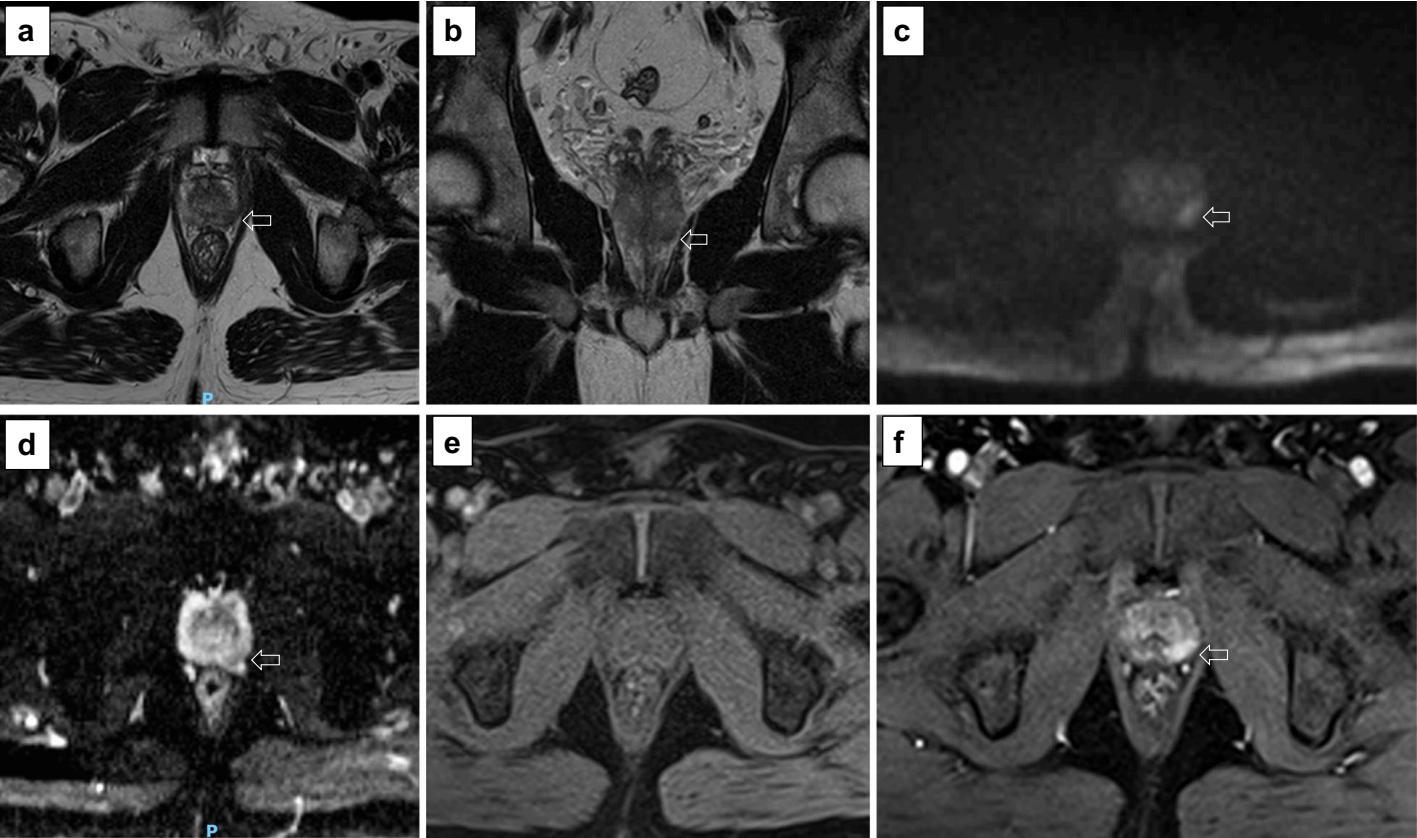
Axial (**a**) and coronal (**b**) T2w imaging, high *b* value (**c**), ADC map (**d**) and pre-contrast (**e**) and DCE acquisitions (**f**) of a study that was given a PI-QUAL score of 3. At least two MR sequences taken together are of diagnostic quality: although T2w imaging is of good diagnostic quality, some parameters are not compliant with the PI-RADS v.2.1 technical recommendations such as the field of view for DWI (23 × 31 cm in **c**, **d**—please note that the DWI images here are magnified) and the temporal resolution (17 s) of DCE sequences. The arrows indicate a lesion (Likert 4/5) in the left peripheral zone between 4 and 5 o’clock. Targeted biopsy revealed Gleason 3 + 4 disease

**Fig. 25:**
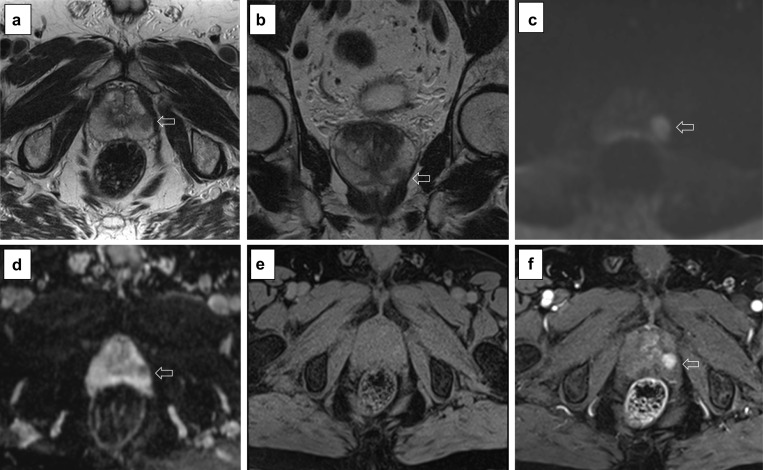
Axial (**a**) and coronal (**b**) T2w imaging, high *b* value (**c**), ADC map (**d**) and pre-contrast (**e**) and DCE acquisitions (**f**) of a study that was given a PI-QUAL score of 4 because the temporal resolution (17 s) of DCE sequences is not compliant with the PI-RADS v. 2.1 technical recommendations, but overall, two or more MR sequences are independently of diagnostic quality. The arrows indicate a lesion (Likert 4/5) in the left peripheral zone at midgland. Targeted biopsy revealed Gleason 4 + 3 disease

**Fig. 26:**
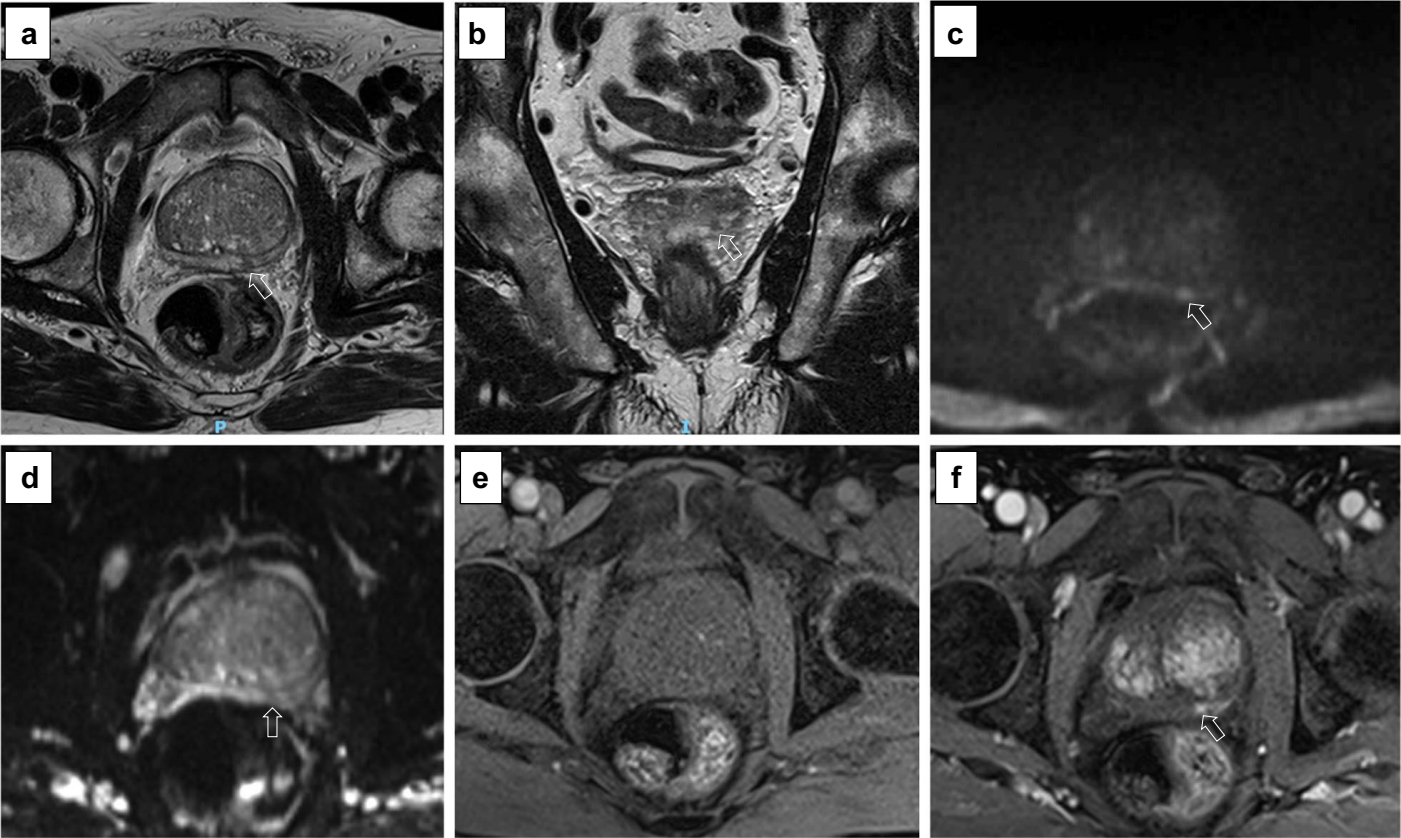
Axial (**a**) and coronal (**b**) T2w imaging, high *b* value (**c**), ADC map (**d**) and pre-contrast (**e**) and DCE acquisitions (**f**) of a study that was given a PI-QUAL score of 5. All MR sequences are of optimal diagnostic quality and fully compliant with PI-RADS v. 2.1 technical recommendations. The arrows indicate a lesion ( Likert 4/5) in the left peripheral zone at 5 o’clock. Targeted biopsy revealed Gleason 3 + 4 disease

- *PI-QUAL 1*: a case of PI-QUAL 1 is shown in Fig. 22.
- *PI-QUAL 2*: a case of PI-QUAL 2 is shown in Fig. 23.
- *PI-QUAL 3*: a case of PI-QUAL 3 is shown in Fig. 24.
- *PI-QUAL 4*: a case of PI-QUAL 4 is shown in Fig. 25.
- *PI-QUAL 5*: a case of PI-QUAL 5 is shown in Fig. 26.

## Conclusions

Adherence to the technical parameters of mpMRI as outlined in the PI-RADS v. 2.1 guidelines [8] is the starting point to improve the quality of prostate MRI.

It is important to understand that a scan performed according to these guidelines may still not be of adequate diagnostic quality. This is usually due to the presence of artefacts or due to poor signal-to-noise ratio (sometimes due to a pressure to reduce scan acquisition time).

In addition to this, the use of rectal enemas, specific dietary restrictions and the administration of anti-spasmodic agents have been shown to improve the quality of prostate MRI as they reduce artefacts due to rectal distension and bowel motility [10, 27, 28].

Newer MR acquisition techniques can be used to improve this (e.g. parallel imaging and motion reduction techniques), and it is of utmost importance to work with MR physicists and radiographers to obtain a set of sequences of the best diagnostic quality for each type of machine. Further studies on what is most important in the technical guidelines for prostate mpMRI are warranted, and these include the creation of a sequence bank for sharing best practice to improve mpMRI quality along with the use of automated methods (e.g. artificial intelligence) [26].

The first version of PI-QUAL is the start of identifying a framework for the assessment of prostate MR quality, but we anticipate that further refinement and prospective validation will be carried out in due course.

## Data Availability

Not applicable.

## Abbreviations

ADC: Apparent diffusion coefficient
DWI: Diffusion-weighted imaging
DCE: Dynamic contrast enhanced
FOV: Field of view
MRI: Magnetic resonance imaging
mpMRI: Multiparametric magnetic MRI
PI-QUAL: Prostate Imaging Quality
PI-RADS: Prostate Imaging-Reporting and Data System
STIR: Short tau inversion recovery
SNR: Signal-to-noise ratio
T2w imaging: T2-weighted imaging

## References

[1] De Visschere PJL, Briganti A, Fütterer JJ (2016). Role of multiparametric magnetic resonance imaging in early detection of prostate cancer. Insights Imaging.

[2] Giganti F, Rosenkrantz AB, Villeirs GM (2019). The evolution of MRI of the prostate: the past, the present, and the future. AJR Am J Roentgenol.

[3] Stabile A, Giganti F, Rosenkrantz AB (2020). Multiparametric MRI for prostate cancer diagnosis: current status and future directions. Nat Rev Urol.

[4] Oberlin DT, Casalino DD, Miller FH, Meeks JJ (2017). Dramatic increase in the utilization of multiparametric magnetic resonance imaging for detection and management of prostate cancer. Abdom Radiol (NY).

[5] Woo S, Hyun C, Youn S, Yeon J, Hyup S, Novara G (2017). Diagnostic performance of prostate imaging reporting and data system version 2 for detection of prostate cancer: a systematic review and diagnostic meta-analysis. Eur Urol.

[6] Barentsz JO, Richenberg J, Clements R (2012). ESUR prostate MR guidelines. Eur Radiol.

[7] Weinreb JC, Barentsz JO, Choyke PL (2016). PI-RADS prostate imaging—reporting and data system: 2015, Version 2. Eur Urol.

[8] Turkbey B, Rosenkrantz AB, Haider MA (2019). Prostate Imaging reporting and data system version 2.1: 2019 update of prostate imaging reporting and data system version 2. Eur Urol.

[9] Suf PS, Sackett J, Shih JH (2021). Quality of prostate MRI: is the PI-RADS standard sufficient?. Acad Radiol.

[10] Purysko AS, Mielke N, Bullen J (2020). Influence of enema and dietary restrictions on prostate MR image quality: a multireader study. Acad Radiol.

[11] Esses SJ, Taneja SS, Rosenkrantz AB (2018). Imaging facilities’ adherence to PI-RADS v2 minimum technical standards for the performance of prostate MRI. Acad Radiol.

[12] Jambor I (2017). Optimization of prostate MRI acquisition and post-processing protocol: a pictorial review with access to acquisition protocols. Acta Radiol Open.

[13] Lim C, Quon J, Mcinnes M, Shabana WM, El-khodary M, Schieda N (2015). Does a cleansing enema improve image quality of 3T surface coil multiparametric prostate MRI?. J Magn Reson Imaging.

[14] Padhani AR, Khoo VS, Suckling J, Husband JE, Leach MO, Dearnaley DP (1999). Evaluating the effect of rectal distension and rectal movement on prostate gland position using cine MRI. Int J Radiat Oncol Biol Phys.

[15] Coskun M, Mehralivand S, Shih JH, Merino MJ, Wood BJ (2020). Impact of bowel preparation with Fleet’s ^TM^ enema on prostate MRI quality. Abdom Radiol (NY).

[16] Caglic I, Barrett T (2019). Optimising prostate mpMRI: prepare for success. Clin Radiol.

[17] Plodeck V, Georg C, Hans R (2020). Rectal gas—induced susceptibility artefacts on prostate diffusion-weighted MRI with EPI read-out at 3.0 T: does a preparatory micro-enema improve image quality?. Abdom Radiol (NY).

[18] Ullrich T, Quentin M, Schmaltz AK, Arsov C, Rubbert C, Blondin D (2018). Hyoscine butylbromide significantly decreases motion artefacts and allows better delineation of anatomic structures in mp-MRI of the prostate European Society of Urogenital Radiology. Eur Radiol.

[19] Ullrich T, Quentin M, Oelers C (2017). Magnetic resonance imaging of the prostate at 1.5 versus 3.0 T: a prospective comparison study of image quality. Eur J Radiol.

[20] Wagner M, Rief M, Busch J (2010). Effect of butylscopolamine on image quality in MRI of the prostate. Clin Radiol.

[21] Brizmohun Appayya M, Adshead J, Ahmed HU (2018). National implementation of multi-parametric magnetic resonance imaging for prostate cancer detection—recommendations from a UK consensus meeting. BJU Int.

[22] De Rooij M, Israël B, Tummers M (2020). ESUR / ESUI consensus statements on multi-parametric MRI for the detection of clinically significant prostate cancer: quality requirements for image acquisition, interpretation and radiologists’ training. Eur Radiol.

[23] Giganti F, Allen C, Emberton M, Moore CM (2020). Prostate imaging quality (PI-QUAL): a new quality control scoring system for multiparametric magnetic resonance imaging of the prostate from the PRECISION trial. Eur Urol Oncol.

[24] Kasivisvanathan V, Rannikko AS, Borghi M (2018). MRI-targeted or standard biopsy for prostate-cancer diagnosis. N Engl J Med.

[25] Giannarini G, Valotto C, Girometti R (2020). Measuring the quality of diagnostic prostate magnetic resonance imaging: a urologist’s perspective. Eur Urol.

[26] Turkbey B, Choyke PL (2020). PI-QUAL, a new system for evaluating prostate magnetic resonance imaging quality: is beauty in the eye of the beholder?. Eur Urol Oncol.

[27] Brennan DL, Lazarakis S, Lee A, Tan TH, Chin KY, Oon SF (2021). Do antispasmodics or rectal enemas improve image quality on multiparametric prostate MRI? An 'evidence-based practice' review of the literature. Abdom Radiol (NY).

[28] Caglic I, Hansen NL, Slough RA, Patterson AJ, Barrett T (2017). Evaluating the effect of rectal distension on prostate multiparametric MRI image quality. Eur J Radiol.

